# Clinical relevance of the severe abnormalities of the T cell compartment in septic shock patients

**DOI:** 10.1186/cc7731

**Published:** 2009-02-25

**Authors:** Jorge Monserrat, Raul de Pablo, Eduardo Reyes, David Díaz, Hugo Barcenilla, Manuel R Zapata, Antonio De la Hera, Alfredo Prieto, Melchor Álvarez-Mon

**Affiliations:** 1 Laboratory of Immune System Diseases and Oncology, National Biotechnology Center – Department of Medicine (CNB-CSIC) Associated Unit, University of Alcalá, Alcalá de Henares, 28871, Madrid, Spain; 2 Intensive Care Unit, Hospital Universitario Príncipe de Asturias, Alcalá de Henares, 28871, Madrid, Spain; 3 Immune System Diseases and Oncology Service, Hospital Universitario Príncipe de Asturias, Alcalá de Henares, 28871, Madrid, Spain

## Abstract

**Introduction:**

Given the pivotal role of T lymphocytes in the immune system, patients with septic shock may show T cell abnormalities. We have characterised the T cell compartment in septic shock and assess its clinical implications.

**Methods:**

T lymphocytes from the peripheral blood of 52 patients with septic shock and 36 healthy control subjects were analysed on admission to the intensive care unit, baseline, and 3, 7, 14 and 28 days later. T cell phenotypes (CD3+CD4+/CD3+CD8+, CD45RA+/CD45RO+, CD62L+/CD28+) were assessed by quantitative flow cytometry.

**Results:**

CD3+, CD3+CD4+ and CD3+CD8+ lymphocyte counts were significantly lower in patients with septic shock than control subjects. In surviving patients, CD3+CD4+ lymphocytes had normalised after 14 days, yet CD3+CD8+ numbers were still low. Non effector CD45RA+CD45RO- subsets of CD3+CD4+ and CD3+CD8+ were persistently low during patient follow up. CD3+CD8+CD28+ and CD3+CD8+CD62L+ were reduced in patients versus controls and survivors versus nonsurvivors in the first three days. A prediction receptor operative curve revealed that for the CD3+CD8+CD28+ subset, a cutoff of 136 cells/ml showed 70% sensitivity and 100% specificity for predicting death and the area under the curve was 0.84 at admission. Corresponding values for CD3+CD8+CD62L+ were 141 cells/ml, 60% sensitivity, 100% specificity and an area under the curve of 0.75.

**Conclusions:**

A severe redistribution of T lymphocyte subsets is found in septic shock patients. A different kinetic pattern of T cell subset involvement is observed in surviving and nonsurviving patients, with lower numbers of circulating CD3+CD8+CD28+ and CD3+CD8+CD62L+ associated with a better disease outcome.

## Introduction

Sepsis has been defined as the systemic inflammatory response syndrome that occurs in response to bacterial infection [1]. Although the pathogenesis of sepsis is complex, current evidence points to a direct triggering effect of bacteria and, in greater measure, to an abnormal systemic immune response [2-4].

The T cell compartment plays a pivotal role in regulating the effector stage of the immune response. CD3+CD4+ T cells are mainly involved in the regulation of the immune response, and CD3+CD8+ T cells are critical in the cytotoxic response [5]. Several molecules, mainly the T cell receptor/CD3 complex and other co-receptors including CD28, contribute to the activation of T lymphocytes. The expression patterns of other molecules, such as the CD45 isoforms RA and RO, vary during the different T cell activation effector stages [6]. Activated T lymphocytes show several profiles (proinflammatory or anti-inflammatory) of cytokine production [7]. Other molecules, such as CD62L, participate in the immune response by regulating the tissue distribution of the T lymphocytes.

A role for T lymphocytes in severe systemic bacterial infections has been described in several studies [13-16] , and the findings of other investigations have supported the notion that T lymphocytes are involved in the pathogenesis of septic shock [8-12]. In this translational study, we have further characterised the abnormalities of the T cell compartment in septic shock and explore its clinical significance. During the first 28 days of follow up circulating T lymphocytes of 52 patients with septic shock admitted to the intensive care unit (ICU) were analysed and 36 healthy subjects were analysed in parallel. We determined the counts and distributions of the main T cell subsets, as well as their stage of activation (CD3, CD4, CD8, CD28, 45RA, 45RO and 62L antigens).

## Materials and methods

### Patients

Fifty-two consecutive patients admitted to the ICU of the University Hospital Príncipe de Asturias, Madrid, Spain, with septic shock, diagnosed according to the criteria of the American College of Chest Physicians/Society of Critical Care Medicine [13], were enrolled in the study. A further requirement was the demonstration of an infectious aetiology through microbiological (Gram stain and/or culture) and/or radiological techniques, or direct observation of the infection focus. The study protocol did not call for a standardised approach to critical care. Exclusion criteria were: anything causing primary or acquired immunodeficiency, previous immunosuppressive or immunomodulation treatment, cancer, or autoimmune or allergic disease. The study was conducted according to the guidelines of the 1975 Declaration of Helsinki, after obtaining the Hospital Universitario Príncipe de Asturias Ethics Committee approval. Written informed consent was obtained from each subject included in the study or surrogate legal representatives.

Thirty-six age-matched and sex-matched healthy blood donors were studied in parallel with the patients (0 and 28 days of the follow up). They were studied to control the adequacy of the cytometric techniques as well as to characterise the normal range of the T lymphocyte compartment parameters analysed.

### Study design

Blood was collected from the patients at baseline (ICU admission) and at 3, 7, 14 and 28 days of follow up, and at baseline and at 28 days in healthy controls. White blood cell differential counts were conducted in a COULTER^® ^LH instrument (Beckman-Coulter Inc, Fullerton, CA, USA).

### Cell separation and *in vitro *culture

Peripheral blood mononuclear cells (PBMC) were obtained from heparinised venous blood by Ficoll-Hypaque (Lymphoprep Nyegaard, Oslo, Norway) density gradient centrifugation. Cells were resuspended (1 × 106 cells/ml) in RPMI-1640 (Biowhittaker Inc, Walkersville, MD, USA) supplemented with 10% heat-inactivated FBS (Cangera International, Ontario, Canada), 25 mM Hepes (Biochrom KG, Berlin, Germany) and 1% penicillin streptomycin (Difco Lab, Detroit, MI, USA).

### Surface immunofluorescence and T cell numbers

T cells were phenotypically analysed in PBMC by four-colour flow cytometry in a FACScalibur cytometer using CellQuest-3.3 software (Becton-Dickinson, San Jose, CA, USA). PBMC were incubated with combinations of fluorescein isothiocyanate (FITC), phycoerythrin (PE), phycoerythrin-cyanine 5.5 (PE-cy5.5), peridinin chlorophyll protein (PerCP) and allophycocyanin (APC)-labelled monoclonal antibodies. The monoclonal antibodies were CD3-PerCP, CD3-FITC, CD45RA FITC, CD56-PE, CD28-PE, CD62L-PE (Becton-Dickinson, San Jose, CA, USA), CD19-PE-CY5, CD8-APC, CD45RO PE (Caltag Laboratories, San Francisco, CA, USA).

### Absolute number lymphocytes calculation

The absolute numbers of T lymphocyte subsets were calculated according to standard flow cytometry criteria for lymphocyte subset identification and the lymphocytes obtained in conventional haemogram. First, we calculated the percentage of cells expressing CD3 in the total lymphocytes gate defined by forward and side scatter in PBMC. The absolute number of circulatory T lymphocytes was calculated by the percentage of CD3+ cells in peripheral blood lymphocytes multiplied by the total number of lymphocytes per microlitre measured by a Coulter^®^. Next, we obtained the absolute number of CD4+ and CD8+ T lymphocytes by multiplying the total number of T lymphocytes previously calculated by the percentage of positive cells for each one of both antigens in CD3+ T cells. We simultaneously stained PBMC with CD3, CD4 and CD8 antibodies to obtain this data. Finally, we calculated the absolute number of the CD3+CD4+ and CD3+CD8+ T cells subsets defined by the expression of CD45 isoforms CD45RA+CD45RO-, CD45RO+CD45RA-, CD45RA+CD45RO+ and the expression of the antigens CD28+ and CD62L+. To calculate these numbers, we multiplied the percentage obtained of each subset in the parents' CD3+CD4+ or CD3+CD8+ populations by the absolute count of CD3+CD4+ and CD3+CD8+ T cells, respectively. All absolute numbers are expressed as cells/ml.

### Statistical analysis

Analyses were performed using SPSS-11.0 software (SPSS, Chicago, IL, USA). Most variables did not fulfill the normality hypothesis, so the Mann Whitney U-test for non-parametric data was used to analyse differences between the groups, and analysis of variance followed by Wilcoxon Signed Ranks tests were used for within group analyses. The level of significance was set at *P* < 0.05.

## Results

### Patients

There were ninety-two ICU admissions with a diagnosis of septic shock during the study period. Forty patients were excluded from the study: three patients were HIV-positive, 18 patients were taking corticosteroids on admission, 12 were on or had received chemotherapy, five patients suffered neoplasia, one had rheumatoid arthritis and one had anaphylactic shock in response to antibiotic therapy for sepsis. Mortality was 34.6%. The mean (± standard error) Acute Physiology and Chronic Health Evaluation (APACHE) II [14] score was 25.3 ± 1.5, the multiple organ dysfunction syndrome (MODS) score [15] was 7.78 ± 0.56 and Sequential Organ Failure Assessment (SOFA) score [16] was 9.09 ± 0.59 at admission to the ICU. All the patients who died did so before day 14. The characteristics of the study patients are summarised in Table 1.

**Table 1:** Clinical characteristics of the patients with septic shock at intensive care unit admission

		Outcome		
			
	Controls (n = 52)	Survivors (n = 34)	Nonsurvivors (n = 18)	p value (Survivor vs nonsurvivor)
Age (years)	62.0 ± 3.4	61.0 ± 3.3	64.5 ± 4.2	0.57
Sex, n (%)*				0.11
Male	36 (69.3)	21 (61.8)	15 (83.3)	
Female	16 (30.7)	13 (38.2)	3 (16.6)	
Lymphocyte (cells/μl)	2095 ± 93	1048 ± 192	1235 ± 178	0.61
		Scores at admission in ICU		
APACHE II		21.64 ± 1.38	29.25 ± 1.50	0.001
MODS		6.65 ± 0.54	9.57 ± 0.99	0.014
δ-MODS		2.25 ± 0.38	3.54 ± 0.73	0.169
SOFA		7.96 ± 0.67	10.64 ± 0.89	0.021
δ-SOFA		1.70 ± 0.47	2.90 ± 0.96	0.34
Pathogen types, n (%)*				0.517
Gram-positive cocci		8 (23.5)	3 (16.7)	
Gram-negative bacilli		6 (17.7)	4 (22.2)	
Polymicrobial disease		3 (8.8)	4 (22.2)	
Unknown, non-detectable		17 (50.0)	7 (38.9)	

Data are number of patients (%) or mean ± standard error of the mean.

* *P* values were determined by bivariate statistical analysis.

APACHE = Acute Physiology and Chronic Health Evaluation; ICU = intensive care unit; MODS = multiple organ dysfunction syndrome; SOFA = Sequential Organ Failure Assessment.

### Surviving and nonsurviving patients with septic shock show different patterns of circulating T cell subsets

Counts and distributions of the main circulating T lymphocyte subsets were systematically examined in 52 patients with septic shock at admission to the ICU and at 3, 7, 14 and 28 days of follow up in the ICU. Furthermore, patients were classified as survivors or nonsurvivors according to their clinical outcome of sepsis during the four weeks of follow up. Thirty-six healthy blood donors who were age-matched and sex-matched (60 ± 3.4 years, 25 men and 11 women) were studied in parallel with the patients as controls of the adequacy of the cytometric technique procedure as well as for characterisation of the normal range of the T lymphocyte compartment parameters analysed.

**Figure 1:**
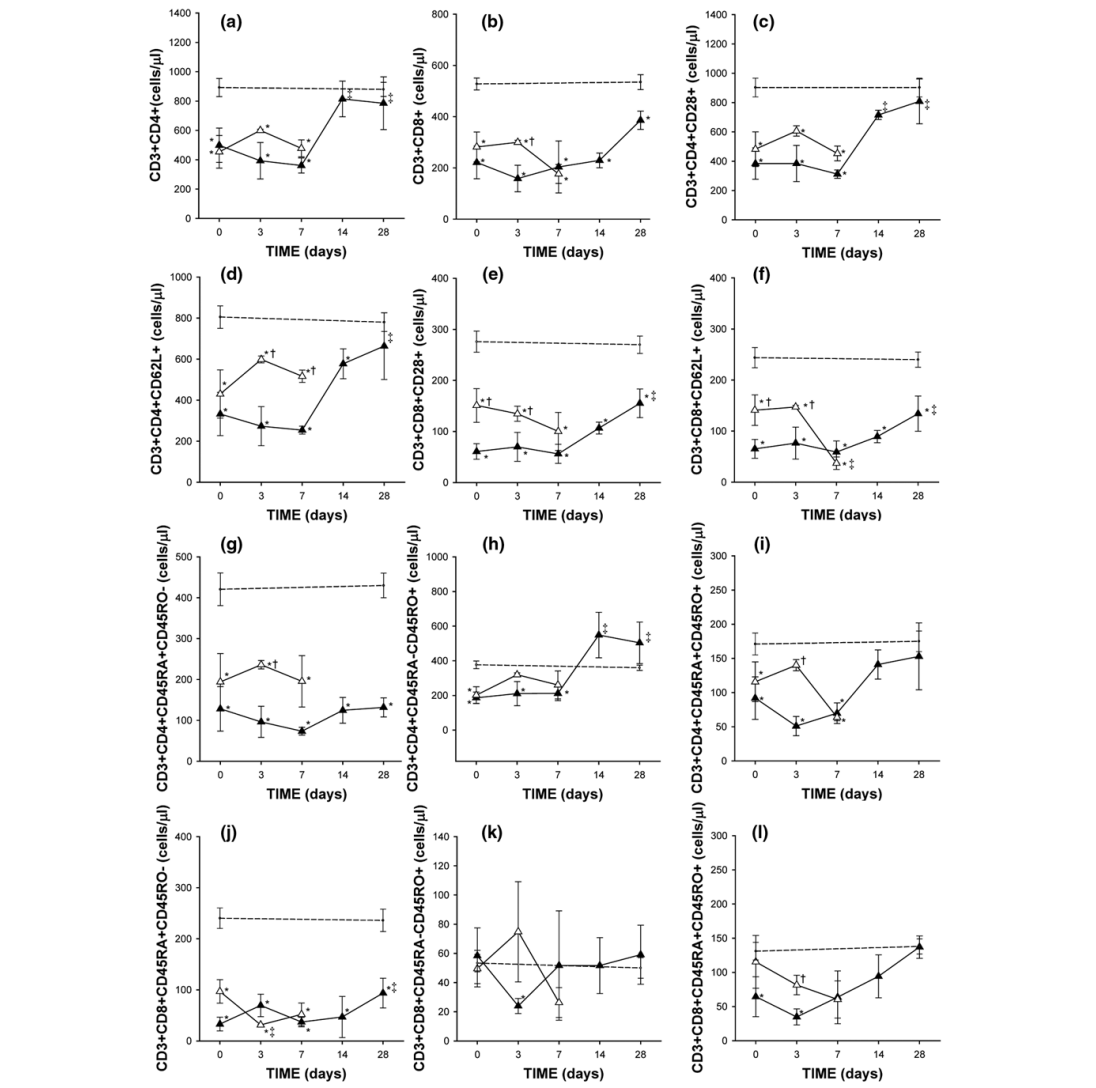
Kinetics of peripheral blood counts of T lymphocyte subsets in patients with septic shock during their stay in the intensive care unit. Data presented as surviving patients (black tirangles) and nonsurviving patients (white triangles). The dotted line represents the mean value recorded in the healthy controls. All values are expressed as the mean number of cells per microlitre ± standard error of the mean. * *P* < 0.05 for survivors or nonsurvivors versus healthy controls; † *P* < 0.05 for survivors versus nonsurvivors; ‡* P* < 0.05 for each follow up time versus baseline or admission to the intensive care unit.

Both surviving and nonsurviving patients showed significantly lower absolute CD3+ T lymphocyte numbers (surviving 740 ± 152 cells/μl, non surviving 746 ± 144 cells/μl respectively) than controls (1394 ± 36 cells/μl) on admission and during the first 14 days of follow up (surviving 7 and 14 days 575 ± 148 cells/μl and 1044 ± 150 cells/μl, respectively, non surviving 7 days 713 ± 70 cells/μl). In survivors, CD3+ counts had significantly returned to normal by day 28 (1191 ± 172 cells/μl). The CD3+CD4+ T cell subset was also reduced in surviving and nonsurviving patients with respect to healthy controls at baseline and during the first week of follow up. However, this dramatically decreased CD3+CD4+ T lymphocyte count had normalised in survivors by day 14 (Figure 1a). On admission, CD3+CD8+ T lymphocytes were also lower in survivors and nonsurvivors versus controls. In survivors, this T cell subset showed a further drop on day 3 of follow up, followed by a gradual recovery, although numbers failed to reach the counts recorded in healthy controls. In addition, CD3+CD8+ T lymphocyte count in survivors was significantly diminished with respect to nonsurvivors on day 3 (Figure 1b).

The activation stage of the CD3+CD4+ and CD3+CD8+ T lymphocytes was determined by examining the expression of the CD45 RA and RO isoforms. The retraction in circulating CD3+CD4+ and CD3+CD8+ T lymphocytes observed in the patients could be mainly explained by a decrease in the noneffector CD45RA+CD45RO- subset (Figures 1g,j). Interestingly, CD3+CD4+CD45RA+CD45RO- and CD3+CD8+CD45RA+CD45RO- T lymphocytes remained low in survivors at the end of follow up. We detected a significant decrease in CD3+CD4+CD45RA+CD45RO- T cells on day 3 with respect to the nonsurviving patients. CD3+CD4+CD45RA-CD45RO+ T cell counts varied over time from a significant reduction during the first week of follow up to elevated numbers in survivors during the last two weeks of the study (Figures 1g,h). Finally, the analysis of effector subsets, characterised by being double positive (CD45RA+CD45RO+) [6,17,18], showed that were significantly reduced in both CD4+ and CD8+ T lymphocyte subsets at baseline, 3 and 7 days in both groups of patients compared with controls and in survivors compared with nonsurvivors at 3 days. From day 7 of follow up onwards, these values normalised in the surviving patients (Figures 1i,l).

**Figure 2:**
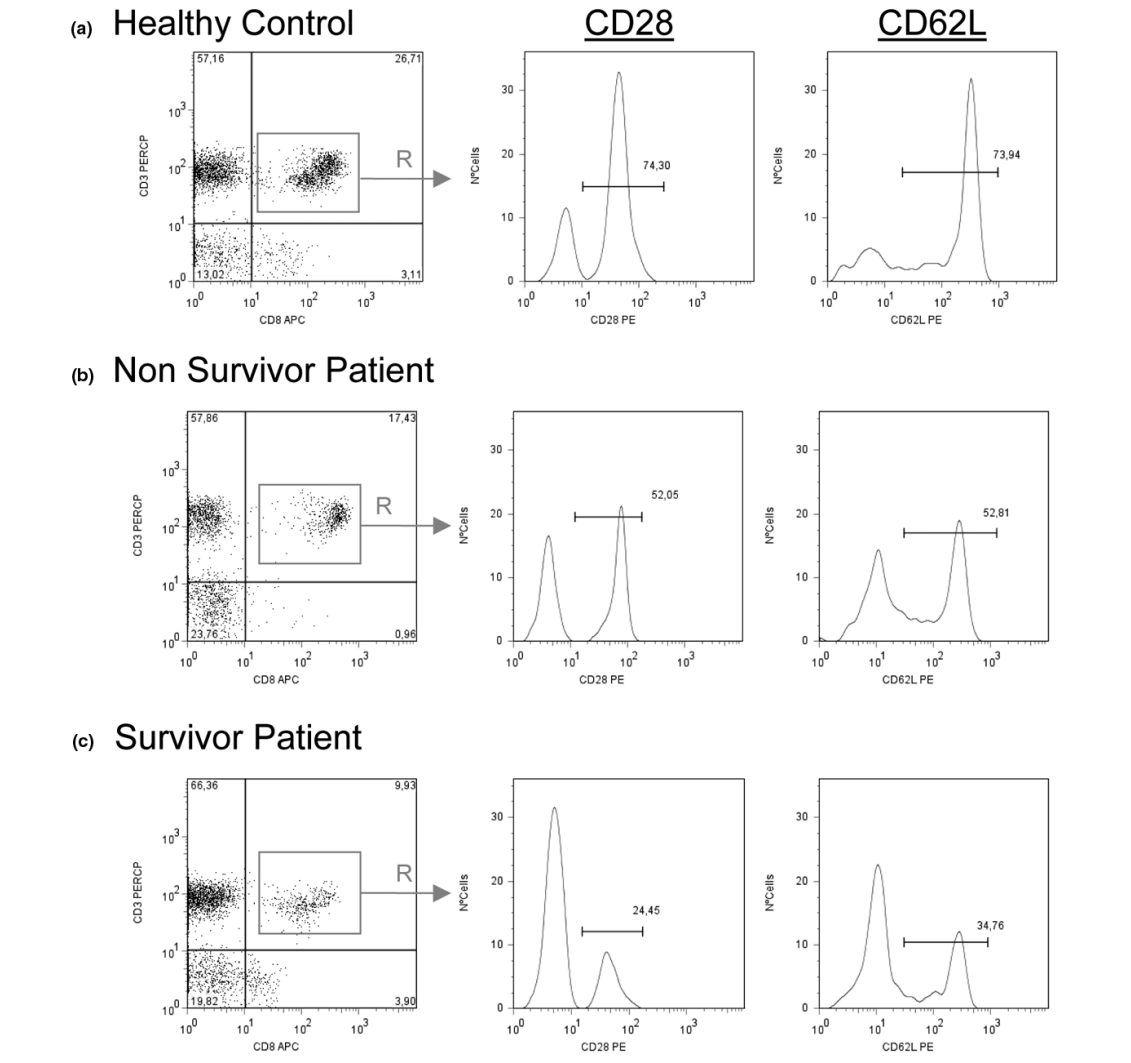
Flow cytometry data analysis of the CD28 and CD62L surface expression in CD3+CD8+ T lymphocytes from peripheral blood of septic shock patients. Panels show CD28 and CD62L expression by CD3+CD8+ gated (Region = R) lymphocytes from peripheral blood of a representative **(a) **healthy control, **(b)** nonsurvivor and **(c)** survivor septic shock patient at the moment of admission to the intensive care unit.

We also analysed the expression of CD28 and CD62L antigens on CD3+CD4+ and CD3+CD8+ T lymphocytes. When CD3+CD8+ T lymphocytes are activated, CD28 expression is lost [19,20]. The number of circulating CD3+CD8+CD28+ T cells was significantly and constantly reduced in patients with septic shock compared with the healthy subjects and in survivors compared with nonsurvivors during the first three days of follow up (Figures 1e and 2). Similar behaviour was shown by CD3+CD8+CD62L+ T cells (Figures 1f and 2). Numbers of circulating CD3+CD8+CD28- T cells were normal in both groups of patients (data not shown).

A prediction receptor operative curve (ROC) was then used to estimate the value of CD3+CD8+CD28+ and CD3+CD8+CD62L+ T cell counts for predicting death in the patients with septic shock at admission and days 3 and 7. We found that a cutoff value of 136 CD3+CD8+CD28+ T cells/ml on admission to the ICU of a patient with septic shock showed a sensitivity of 70% and 100% specificity for predicting the risk of death, and the area under the ROC curve was 0.84. For the CD3+CD8+CD62L+ T cells, the cut off on admission was 141 cells/ml, with a 60% sensitivity and 100% specificity for predicting the risk of death and an area under the curve of 0.75. The sensitivity and specificity of the data obtained at days 3 and 7 were worse than those found at admission (data not shown).

The number of circulating CD3+CD4+CD28+ T cells was significantly lower in surviving and nonsurviving patients with septic shock compared with healthy controls on admission and on day 3 of follow-up (Figure 1c). In survivors, this was followed by a gradual recovery of CD3+CD4+CD28+ T cell numbers during the course of follow up. CD3+CD4+CD62L+ T cells showed a similar pattern of behaviour (Figure 1d). In the parallel study performed in healthy blood donors (at 0 and 28 days of the follow up), no significant variations in the absolute counts and distribution of the different subsets of T cells analysed were detected.

## Discussion

In this study, we show that surviving and nonsurviving patients with septic shock have different patterns of involvement in circulating T lymphocyte compartment. A drop in circulating CD3+CD4+ and CD3+CD8+ T cells has been described in patients with severe sepsis or septic shock at admission to the ICU [8-12]. In our kinetic study, we also observed that this T lymphopenia persists during the first week of follow up and is independent of the outcome. Moreover, by the end of the second week of follow up, the absolute number of circulating CD3+CD4+ T cells had clearly normalised. In contrast, after four weeks of follow up, there was still no return to normal circulating numbers of CD3+CD8+ T cells.

In a mouse sepsis model of caecal ligation and puncture, the depletion of CD3+CD8+ T and natural killer cells was associated with a survival benefit with decreased blood bacterial concentrations, improved physiological function and an attenuated proinflammatory response [21]. It has been reported that mice infected with *Plasmodium berghei *develop a syndrome similar to septic shock and the depletion of CD3+CD8+ T cells also significantly ameliorates the complications that induce shock [22]. In addition, in patients with trauma and multiple organ failure, nonsurvivors showed CD3+CD8+ T cell numbers that were two-fold those recorded in survivors [23]. In agreement with these experimental and clinical findings, we observed significantly lower CD3+CD8+ T cell counts in survivors compared with nonsurvivors on day three of follow up. Thus, diminished circulating CD3+CD8+T cells might have a protective pathogenic role in the outcome of septic shock.

CD45 is a phosphatase that is essential in T cell development and antigen receptor signalling [24]. The expression patterns of the RA and RO isoforms of CD45 by T lymphocytes serve to identify subsets associated with different stages of T cell activation [6]. When noneffector CD45RA+CD45RO- T lymphocytes are activated by inflammatory agents, such as bacterial infection, CD45RO is up-regulated and CD45RA down-regulated [6]. Thus, our patients showed a persistent reduction in circulating noneffector CD4+CD45RA+ CD45RO- and CD8+CD45RA+ CD45RO- T cells. In contrast, numbers of CD8+CD45RA-CD45RO+ T cells remained normal and CD4+CD45RA-CD45RO+ T cell counts initially fell yet had returned to normal by the second week of follow up in surviving patients. An increased percentage of CD3+ cells expressing CD45RO has been reported in patients with sepsis [25]. Our findings could be the consequence of abnormal polyclonal activation of circulating T lymphocytes. It has been proposed that the switch of T cells from a CD45RA+CD45RO- to a CD45RA-CD45RO+ phenotype may have a functional effect in halting the sustained immune response in an effort to avoid tissue injury [6]. Thus, it is possible to suggest that the expansion of CD4+CD45RA-CD45RO+ T lymphocytes observed here during the follow up of surviving patients might be considered a compensatory anti-inflammatory mechanism that develops in these patients with septic shock.

CD28 is a costimulatory molecule that plays a key role in regulating the activation and survival of T lymphocytes [26]. Activation of CD3+CD8+ T lymphocytes has been related to the loss of CD28 expression [19,20]. In effect, it has been reported that patients with severe sepsis showed a significant reduction in T lymphocyte CD28 expression [27]. Our data indicate diminished circulating CD3+CD8+CD28+ T cell numbers in patients with septic shock with respect to healthy subjects on admission to the ICU and at least during the first 28 days of follow up. Interestingly, a reduced CD3+CD8+CD28+ T cell count during the first three days of admission to the ICU was of prognostic value for predicting the survival of a patient. Conversely, an elevated number of circulating CD3+CD8+CD28+ T cells was associated with a worse prognosis for the patient. Recently, it has been reported that the stimulation of CD28 by a monoclonal antibody in healthy volunteers is followed by severe multiple cytokine-release syndrome [28]. Our results support a relevant role for CD3+CD8+CD28+ T cells in the pathogenesis of septic shock. Future studies should address the potential clinical relevance of this cell variable.

The migration of circulating T lymphocytes to peripheral lymph nodes depends on the expression of the CD62L homing receptor [29]. We found here that the down-regulation of L-selectin expression on CD3+CD8+ cells in patients with septic shock was associated with a better outcome. Hence, the rapid migration of CD8+ T cells to peripheral lymph nodes may be a mechanism contributing to patient survival.

Our T lymphocyte phenotype data show a time difference, or shift, in the recirculation of T lymphocytes between patients who survive septic shock and those who do not. Taken together and analysing the phenotype of the circulating T cells according to the activation criteria (CD45RA+ and CD45RO+) related to CD28 (activation and co-stimulation) and CD62L (activation and migration) expression point to a slower migration of naive and effector cells in nonsurviving patients. This different T lymphocyte kinetics would mean a delayed tissue response that could determine the failure of the immune system and the fatal prognosis of the patient. In particular, the delay in the disappearance of CD45RA+, CD45RA+CD45RO+, CD28+, CD62L+, T CD4+ and CD8+ lymphocytes observed between days 3 and 7 of follow up in the nonsurvivors appears to be crucial to the final outcome. Accordingly, in surviving patients, effector cells would migrate more rapidly to tissues and this would in turn trigger the quick action of the immune system in combating the infection and thus determine the survival of the patient. It is known that cellular immune responses play a critical role in the defense against viral infections and strong T-cell responses have been reported in patients who clear infection [30]. If the immune response is late or less efficient against microorganism viral epitopes, the outcome of the disease worsens [30-33].

Not surprisingly, the survivor group had lower APACHE II, MODS and SOFA scores than nonsurvivors. An increasing APACHE II score reflects an increasing severity of illness and escalating risk of hospital death for multidiagnostic ICU patient groups. However, an APACHE II score cannot be directly equated with a specific risk of lower mortality than the same score for a patients with septic shock [34]. In this group of patients, we found that a cut-off value of 136 cells/ml for CD3+CD8+CD28+ T cells and 141 cells/ml for CD3+CD8+CD62L+ T cells on ICU admission showed high specificity for predicting the risk of death. However, it is known that the positive predictive value for APACHE II for the validation study population was only 69.6% and the negative predictive value was 87.9% [35]. Moreover, SOFA, MODS and APACHE II scores require at least 24 hours of monitoring to be performed and lymphocyte phenotyping can be performed in a short time (approximately 2 hours). It is not possible to replace clinical score in septic shock patients by immunological markers. However, these analytical parameters may help to make clinical decisions in these patients and to establish new potential therapeutic targets. Future studies will need to study in more depth the mechanisms involved in the severe abnormality found on the T cell compartment in patients with septic shock.

## Conclusions

Septic shock patients show a severe redistribution of circulating T lymphocyte subsets. We found that CD62L and CD28 expression on circulating T cells at ICU admission are good markers to predict the outcome of shock septic patients. T lymphocyte phenotype data show a time difference in the recirculation of T cells between survivors and nonsurvivors that might provoke a delayed tissue response of the immune system.

## Key messages

• Septic shock patients show a severe redistribution of circulating T lymphocyte subsets.

• CD62L and CD28 expression on circulating T cells at ICU admission are good markers for predicting the outcome of shock septic patients.

• T lymphocyte phenotype data show in nonsurviving patients a slower migration of naive and effector cells that might provoke a delayed immune response.

## Abbreviations

APACHE: Acute Physiology and Chronic Health Evaluation; APC: allophycocyanin; FBS: fetal bovine serum; FITC: fluorescein isothiocyanate; ICU: intensive care unit; MODS: multiple organ dysfunction syndrome; PBMC: peripheral blood mononuclear cells; PE: phycoerythrin; PerCP: peridinin chlorophyll protein; PE-cy5.5: phycoerythrin-cyanine 5.5; ROC: receptor operative curve; SOFA: Sequential Organ Failure Assessment.

## Competing interests

The authors declare that they have no competing interests.

## Authors' contributions

JM and RP are joint authors and contributed equally to this manuscript. AP, MAM, JM and RP contributed to the design of the study and drafted the manuscript. JM, DD and HB obtained the data. JM, RP, MÁ, AH, MRZ and ER participated in data analysis and interpretation of the results.
